# Application of methylene blue for the prevention and treatment of COVID-19: A narrative review

**DOI:** 10.22038/IJBMS.2024.71871.15617

**Published:** 2024

**Authors:** Elaheh Emadi, Daryoush Hamidi Alamdari, Davood Attaran, Soroush Attaran

**Affiliations:** 1 Vascular and Endovascular Surgery Research Center, Mashhad University of Medical Sciences, Mashhad, Iran; 2 Surgical Oncology Research Center, Mashhad University of Medical Sciences, Mashhad, Iran; 3 Department of Biochemistry, Faculty of Medicine, Mashhad University of Medical Sciences, Mashhad, Iran; 4 Lung Diseases Research Center, Mashhad University of Medical Sciences, Mashhad, Iran

**Keywords:** COVID-19, Methylene blue, Methylthioninium chloride, Prevention, Rescue therapy, SARS-CoV-2

## Abstract

The newest virus from the SARS family of viruses called acute syndrome-coronavirus-2 (SARS-CoV-2), which causes COVID-19 disease, was identified in China at the end of 2019. In March 2020, after it spread to 29 additional countries, it was declared a pandemic by the World Health Organization (WHO). SARS-CoV-2 infection mainly starts through the respiratory tract and causes a wide spectrum of symptoms from asymptomatic infections to acute respiratory distress syndrome with multi-organ failure and vasoplegic shock. Among the many immunomodulatory and antiviral drugs that have been studied for the treatment of COVID-19, methylene blue (MB) may play an influential role. This article reviews the history of MB applications, the antiviral effects of MB against SARS-CoV-2, and the results of* in vivo* and *in vitro* studies of the use of MB in COVID-19. Based on studies, MB can simultaneously affect most of the host’s harmful responses caused by SARS-CoV-2 infection due to its multiple properties, including anti-hypoxemia, anti-oxidant, immune system modulator, and antiviral. The use of MB is associated with a reduction in the possibility of getting infection, and mortality, and can be used as a safe, effective, cheap, and available treatment option with minimal side effects for the clinical management of COVID-19.

## Introduction

During the last two decades, diverse coronavirus species that lead to respiratory tract lesions have been identified (1). B814, the first human coronavirus, was discovered in 1965. In 2002–2003, a strain of coronavirus that causes a new respiratory illness called acute respiratory syndrome (SARS) was recognized with a mortality rate of 9%, and based on phylogenetic studies, bats were the origin of the entrance of this virus into the human population. In 2004 and 2005, two new strains, HCoV-NL63 and HCoV-HKU1, were diagnosed in an infant with bronchiolitis in the Netherlands and a patient with pneumonia in Hong Kong, respectively, which did not cause new epidemics in the human population. However, in 2012, the Middle East respiratory syndrome coronavirus (MERS-CoV) isolated from a pneumonia patient in Saudi Arabia was distributed to 21 countries with a mortality rate of approximately 40%. In December 2019, a novel beta virus, SARS-CoV-2 was discovered in Wuhan, Hubei Province, China which is the causative agent of human coronavirus disease 2019 (COVID-19). Although SARS-CoV-2 is less lethal than MERS-CoV, it has a higher transmission rate. In March 2020, COVID-19 was declared a pandemic by the World Health Organization (WHO), covering all continents except Antarctica. SARS-CoV-2s are medium-sized (30 kb) single-stranded RNA viruses with a 5′-cap structure and a 3′ terminal poly-adenylated tail in lipid envelopes with club-like spike protein projections on the surface (2-5). These viruses use the protein-protein interaction (PPI) of the spike glycoprotein (S-protein) with angiotensin-converting enzyme 2 (ACE2) of the host cell surface for entry (3). These spherical virions with a diameter of about 125 nm, an infectivity rate (R_0_) from 1.5 to 6, and an incubation period of 14 days, cause a wide spectrum of symptoms from asymptomatic infections to acute respiratory distress syndrome (ARDS), with multi-organ failure and vasoplegic shock (2, 5, 6). SARS-CoV-2 infection generally starts in the respiratory tract and can be accompanied by clinical problems such as bilateral pneumonia, marked immunopathological changes, and multi-organ failure (mainly lung, heart, brain, kidney, liver, and small intestine) (7). The last update of WHO on 1 December 2023 verified that there have been 698,563,428 confirmed cases of COVID-19, including 6,944,971 deaths. About fourteen percent of infected people suffered from very severe COVID-19 and needed to be hospitalized, and four percent died (8). This makes COVID-19 one of the unprecedented global health challenges that had devastating social and economic effects around the world. Prevention and early treatment of COVID-19 could prevent its harmful effects and improve clinical outcomes (5). Various drugs and compounds have been assessed worldwide (9). Currently, oxygen support, antiviral agents, anticoagulants, anti-oxidants, antibiotics, immunomodulatory drugs (anti-cytokines), fluid therapy, and bradykinin antagonists are used in the clinical management of patients with COVID-19 (10). Most antiviral and anti-cytokine drugs have low effectiveness in ARDS cases with multiple organ failure (11). Because they inhibit the production of a small number of cytokines and do not target the uncontrollable production of free radicals (ROS and RNS) and kinins that play an important role in the inflammatory cascade. Therefore, there was a need for a suitable drug candidate that could inhibit most cytokines, free radicals, and kinin (7, 11). Methylene blue, in addition to all the necessary medicinal properties mentioned, is an FDA-approved, widely available, affordable drug with limited side effects, and for this reason, it is an appropriate drug for the treatment of COVID-19 (9, 12-14). 

Methylene blue has also recently gained attention for its potential preventive role in COVID-19 (2). The key mechanisms by which methylene blue may exert its preventive effects are its ability to inhibit SARS-CoV-2 virus replication and cell protection from viral-induced damage by improving mitochondrial function (3, 7, 11, 15-17). Given the promising preliminary studies on the possible preventive and therapeutic role of methylene blue in COVID-19, more research and clinical trials are needed to prove its efficacy and safety.

**Search Method**

The Google Scholar and PubMed databases were explored to discover the published studies in English addressing the preventive and therapeutic effect of methylene blue in covid 19, both in *in vitro* and clinical trials, without a time limit. The search terms included “coronavirus disease 2019”, “COVID-19”, “SARS-CoV-2”, “methylene blue”, “methylthionine chloride”, “treatment”, and “prevention”.

**Methylene blue (MB or MeBlu)**

***History of methylene blue***

In 1876, methylthionine chloride (methylene blue), a tricyclic phenothiazine molecule, was synthesized through the reaction of a mixture of N, N-dimethyl-p-phenylenediamine (C_8_ H1_2_ N_2_) and hydrogen sulfide (H_2_S) with iron chloride (FeCl_3_) by a German chemist named Heinrich Caro as an aniline-based dye for textile dyeing (2, 18). In 1882, Robert Koch and Paul Erich used MB to stain tuberculosis microorganisms as well as to differentiate the variant types of white blood cells, and therefore, MB entered the field of medicine (2, 4). Since the 1890s, MB has been utilized as a local anesthetic agent due to its sensory nerve-ending blocking property (4). In 1891, its ability to target the malaria organisms was identified by Paul Ehrlich; that was the beginning of its therapeutic application, but its use was stopped due to its unavoidable unfavorable effects (reversible bluish coloration of urine, sclera, and skin) (2, 4, 9, 11, 19, 20). Then, it was employed in 1897 to treat gonorrhea and in 1908 to treat fever (21, 22). MB (oxidant, dark blue) has a reduction potential similar to the oxygen reduction potential. In the cell’s electron transport chain and the in plasma, by accepting electrons on the thiazine ring, during a reversible reaction it is reduced to leucomethylene blue (leucoMB, LMB, MBH_2_, a neutral lipophilic MB radical, a colorless anti-oxidant) (2, 23, 24). Because of this feature, it was exploited as an antidote for cyanide poisoning in the 1920s (25), and in the early 1930s, it was proven to be effective in the treatment of methemoglobinemia, and nowadays it is one of its main uses (26, 27). MB is also a recommended treatment for hypotension in sepsis and vasoplegia (28). In addition, since 1991, evidence has been provided that shows photoactivated MB is effective in removing hepatitis C virus, human immunodeficiency virus (HIV), MERS-CoV, Ebola Virus (EBOV), Nipah virus (NiV), and Crimean–Congo hemorrhagic fever virus (CCHFV) in blood products, which is a new path in antiviral treatment and inactivation of pathogens in plasma (9, 19, 29-32). MB embeds within the nucleic acid structure and the light application stimulates it to produce highly reactive singlet oxygen that oxidizes guanosine and damages the nucleic acid strands (9, 31). Antiviral and antimicrobial effects of MB *in vivo* and *in vitro* have been also demonstrated even in the absence of UV-induced activation at low micromolar concentrations (1, 3, 13, 19, 31, 32). Today, the therapeutic and diagnostic use of MB in diseases such as Alzheimer’s disease, depression, anxiety, psychosis, cancer, prevention of urinary tract infections, detection of joint capsule disruption, guided debridement, lower back pain, oral lichen planus pruritus ani, oral mucositis, and neuroinflammation at the microglial level is being evaluated and used (2, 4, 8, 31-36). With a history of more than 140 years and a wide range of functions, MB is one of the most common safe and FDA-approved drugs. However, it is contraindicated in pregnant and lactating women, in cases with glucose-6-phosphate dehydrogenase (G6PD) deficiency, people with sensitivity to thiazine dyes, and antidepressant drug users (19, 37-40). 

Also, MB has dose-dependent toxicity in doses higher than 7 mg/kg (i.e., >500 mg). Recently, studies have confirmed the effectiveness of MB by oral, inhalation, and injection in the treatment of COVID-19 (8, 13, 23, 31).

***Antiviral effects of methylene blue against SARS-CoV-2***

The possible mechanisms and biological targets involved in SARS-CoV-2 infection that are targeted by methylene blue to improve the symptoms of patients are as follows.

**Figure 1:**
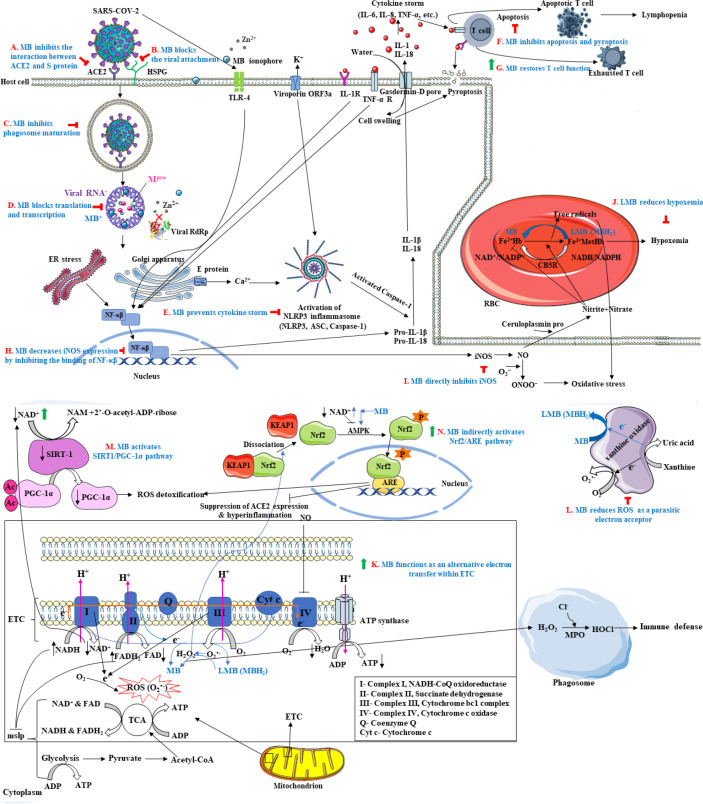
Schematic diagram of the antiviral effect of methylene blue (MB) against SARS-CoV-2

1) The entry process of the virus: the protein receptor in the body called angiotensin-converting enzyme 2 (ACE2) is critical for SARS-CoV-2 cell entrance. SARS-CoV-2 uses the receptor binding domain (RBD) of spike (S) glycoprotein anchored in the viral membrane to bind to this receptor. The human Alveolar and nasal epithelial cells highly express ACE2 receptors. For this reason, SARS-CoV-2 mainly enters the human body through the respiratory tract and first affects the lungs. Because ACE2 receptors are also present on the vascular endothelium lining of all organs, the local pulmonary infection may progress into a systemic immune response and pathology (systemic inflammatory response syndrome (SIRS)). Blocking the interaction of the SARS-CoV-2 S protein and the ACE2 receptor can reduce the virulence potential. Methylene blue can block this interaction with a concentration-dependent mechanical effect. In other words, it competitively occupies the necessary cellular sites for attachment and penetration of the virus (Figure 1 A) (3, 7, 11, 15, 16). Moreover, the positively charged S proteins of SARS-CoV-2 initially attach to the negatively charged cell surface heparan sulfate proteoglycans (HSPGs) and facilitate the interaction between ACE2 and SARS-CoV-2. The cationic methylene blue molecules accumulate on HSPGs and exhibit a shielding effect. This effect prevents viral attachment as well as the subsequent internalization process (Figure 1 B) (13). 

2) The phagosome maturation process: the endosomes play a vital role in the contamination of SARS-CoV-2, and an acidic media (PH<5) is essential for their function and maturation. The neutral and reduced form of methylene blue (leuco-MB) is a lipophilic radical with a weak base (pKa ~9) that could easily pass through the endosome membrane and transiently alkalizes the pH of its cytosolic space. This increase of endosomes’ intracellular pH may further assist in decontaminating the virus due to the endosome maturation inhibition at intermediate stages of endocytosis. Thus, the virions could not be imported into the cytosol (Figure 1 C) (4, 23, 27). 

3) The translation and replication process: when the virions enter the cytosol by endocytosis, the process of their RNA replication initiates with ORF 1a and 1b translation into polyproteins (pp1a and pp1ab). Methylene Blue permeates through the cell membrane, and due to its positive charge and planar tricyclic heteroaromatic structure, intercalates in the negatively charged viral RNA and blocks the translation and replication of the viral RNA (4, 13). In addition, since methylene blue is a Zn^+2^ ionophore, it inhibits the elongation step of RNA-dependent RNA polymerase (RdRp) (RNA replicase), thus aiding viral detoxification (11, 41). The SARS-CoV-2 main protease (M^pro^), the result of proteolysis of pp1a and pp1ab, is necessary to replicate the genome RNA by participating in the non-structural proteins (NSPs) production and virion maturation. Methylene blue by binding to M^pro^ would inactivate it (6, 42) (Figure 1 D). 

4) Cytokine storm or hyperinflammatory syndrome: following SARS-CoV-2 infection, inflammatory signaling pathways are activated and produce large amounts of inflammatory molecules and inflammatory mediators (like IL-6, IL-1, IL-2, IL-10, IL-12, IL-18, TNF-α, IP-10, MCP-1, MIP-1α, etc.), called a cytokine storm. This uncontrolled increase in immune responses leads to tissue damage (6, 11, 16). One of these pathways is NF-kβ which is activated in various cells such as kidney, lung, and liver macrophages, cardiovascular, central nervous, and gastrointestinal cells (43), and is induced due to the SARS-CoV-2 pathogens in two ways, endoplasmic reticulum (ER) stress-induced NF-kβ activation and TLR-mediated NF-kβ activation (44). The SARS- CoV-2 viroporin ORF3a and E protein activate K^+^ efflux at the plasma membrane to the extracellular space and Ca^2+^ influx to the cytosol from Golgi storage to promote the activation of the NLRP3 inflammasome complex (a Nod-like receptor (NLR), the adapter apoptosis-associated speck-like (ASC) protein, and Caspase-1) (ER stress-induced NF-kβ activation). This complex can contribute to the inflammatory responses by stimulating and secreting proinflammatory cytokines IL-1β/IL-18 production and activating caspase-1 (45). The E protein can also prompt macrophages to secrete the inflammatory cytokine pro-IL-1β by its ion-channel activity (46). A cytokine storm occurs following uncontrolled SARS-CoV-2 replication and constant and improper inflammasome signaling in severe COVID-19 patients. The Gasdermin-D pores are created for IL-1β and IL-18 secretion and water influx by the inflammasome activation. Pyroptosis occurs as a result of cell swelling (11, 27). Moreover, after SARS-CoV-2 enters into the host cell, viral RNA activates the Toll-like receptors (TLRs) such as TLR4, TLR3, and TLR7/8. These receptors lead to NF-kβ pathway activation (TLR-mediated NF-kβ activation). Excessive NF-kβ activation starts proinflammatory cytokine production and a cytokine storm (47). Methylene Blue weakens the NF-κβ signaling by reducing the secretion of caspase-1, IL-1β, as well as ASC aggregation, and NLRP3 promoter activity, and subsequently prevents the formation of NLRP3 complex, the cytokine storm, and its consequences (27) (Figure 1 E). MB also helps reduce inflammation by inhibiting the protein-protein interactions of CD40–CD40L and TNF-R–TNFα (data not shown) (31). 

5) Lymphopenia state (decrease in lymphocytes): lymphopenia has been reported in COVID-19 patients (48). High levels of cytokines (IL-1β and TNF-α) or cytopathic effects (change in the structure of virus-infected cells) lead to cell death in T lymphocytes (27). Pyroptosis, a form of cell death, is started by inflammatory signals and a high level of IL-1β (45). Apoptosis, a form of programmed cell death through activating caspases, is also initiated in lymphocytes by TNF-α binding to the TNF-α receptor (TNFR) (49, 50). MB can protect immune cells from death associated with toxic inflammation by inhibiting caspase-1 and caspase-3 (27) (Figure 1 F). Furthermore, it has been reported that methylene blue aids in restoring the ability of the exhausted CD8 T cells for proliferation, cytotoxicity, and cytokine secretion by blocking their downstream inhibitory protein-protein interactions (Figure 1 G) (23, 51). 

6) Nitric oxide synthase (NOS): nitric oxide synthase converts L-arginine to nitric oxide (NO^•^) in endothelial and epithelial cells as well as activated macrophages (4). In SARS-CoV-2 infection, macrophage inducible nitric oxide synthase (iNOS) is induced two or three times more than the normoxic conditions and releases plenty of NO^•^ (16). The ceruloplasmin protein immediately oxidizes NO to nitrite (NO_2_^−^) and nitrate (NO_3_^−^) in the blood. The nitrite crosses through the red blood cell membrane and oxidizes hemoglobin (Hb) to methemoglobin (MetHb), it also degrades Hb, leading to increased hypoxemia (16). NO at high levels, in a perturbation of homeostatic balance, can induce oxidative/nitrosative stress and inflammation as well. For instance, NO can react with superoxide anions (O_2_^−^) (a type of reactive oxygen species) to form toxic peroxynitrite (ONOO^−^) which damages cells and triggers apoptosis (52, 53). 

It is documented, that MB can reduce the adverse effects of nitrite and NO by directly inhibiting iNOS enzymatic activity (Figure 1 I) (2, 16) and decreasing its expression by changing the transcription factors’ binding affinity (NF-κB and STAT1) on the promoter gene of iNOS (Figure 1 H) (54). 

7) Methemoglobinemia state (MetHba, oxygen saturation drop) is one of the clinical problems associated with SARS-CoV-2. Methemoglobin (MetHb) formation occurs when the iron in hemoglobin (Hb) is oxidized from the ferrous state (Fe^2+^) to the ferric state (Fe^3+^), which is related to a decrease in the oxygencarrying capacity (55). In normal conditions, the body struggles to maintain the level of methemoglobin to a minimum with a reduction process through NADH-dependent cytochrome b5 reductase (CB5R, methemoglobin reductase) in red blood cells (RBCs). In case of excessive production of methemoglobin, the natural homeostatic mechanism of the body is unable to neutralize the excessive production of methemoglobin, which ultimately leads to hypoxemia and reduced oxygenation to cells and tissues (55). During SARS-CoV-2 infection, the increased level of nitrite metabolite causes an increase in hemoglobin oxidation to methemoglobin and subsequently, hypoxia (Figure 1 J) (18). 

8) Perturbation in cellular respiration: cellular respiration contains three main stages, glycolysis, citric acid cycle (CAC) or tricarboxylic acid cycle (TCA), and oxidative phosphorylation by which glucose is broken down to produce ATP. 

Under ordinary conditions, ATP is synthesized during the mitochondrial oxidative phosphorylation process of the electron transport chain (ETC) from the oxidation of NADH and FADH_2_ and the electron transfer to the components of the chain and H^+^ gradient along with the reduction of oxygen to water (17). Cytochrome c oxidase (CcO, complex IV, the last enzyme of ETC) catalyzes the cytochrome c oxidation and the oxygen reduction to water. During these processes, ATP is alternately oxidized and reduced, and nitric oxide and oxygen compete to bind to it. In the hypoxia situation induced by SARS-CoV-2 infection, it is mainly in its reduced state and NO binds to it in competition with oxygen (56). The function of CcO is stopped and other components of ETC are also blocked. As a result, the amount of NADH increases relative to NAD^+^. Since mitochondrial substrate level phosphorylation (mSLP) is regulated by the level of NAD, a decrease in NAD and an increase in NADH stop it (27). Hence, pyruvate, the product of glycolysis, is not converted to acetyl-CoA and is reduced in the alternative pathway by the lactate dehydrogenase enzyme to lactic acid. Cytosolic pH decreases and lactic acidosis occurs (57). Moreover, some electrons from complexes I and III are directly transferred to O_2_, which is reduced to the superoxide (O_2_^•−^) (17, 58). This results in an increased formation of reactive oxygen species (ROS) and activation of some signaling pathways such as HIF, NF-κB, AMPK, and cGMP-dependent pathways. The redundant ROS can lead to cell damage and even cell death (56). Methylene blue as a coenzyme (an alternative electron transporter) changes the electron transfer pathway as an alternative electron transporter and helps to regenerate NAD^+^ and cellular respiration (formation of acetyl-CoA from pyruvate instead of lactic acid) by receiving electrons from NADH through complex I (17). After receiving the electron, MB is reduced to leuco-MB. Then, it can donate the electron to CcO and return to its oxide form (MB) (59). Furthermore, the leuco-MB, as a free radical scavenger, can interact with the radical superoxide (O_2_^•−^) generated due to the blockage of complexes I and III, and produce hydrogen peroxide (H_2_O_2_) and reduce ROS (59). The resultant H_2_O_2_ is catalyzed to the hypochlorous acid (HOCl) by the enzyme myeloperoxidase (MPO) which can promote the biocidal action against SARS-CoV-2, especially in neutrophils and phagocytes (Figure 1 K) (60, 61). 

In addition, MB can weaken the formation of ROS by accepting electrons during the function of xanthine oxidase (XO), an enzyme that produces reactive oxygen species (36). In normal situations, XO catalyzes the oxidation of xanthine to uric acid and during this reaction, O_2_^•−^ is also produced from the reduction of oxygen molecules. When MB is present, it accepts electrons and arrests the oxygen molecule reduction (Figure 1 L) (62). 

**Figure 2:**
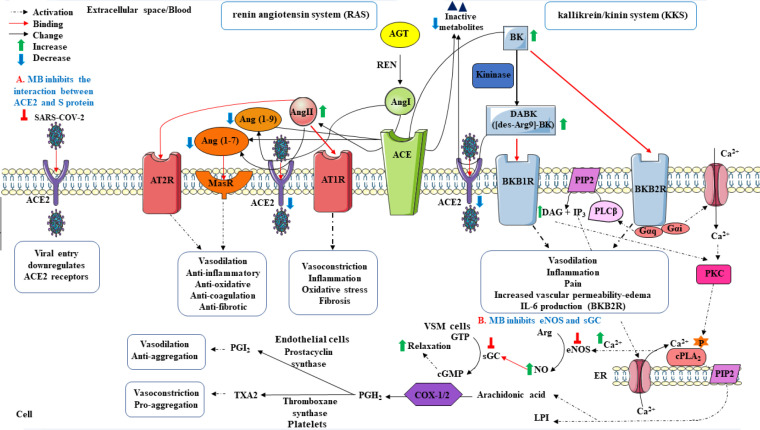
Impacts of methylene blue (MB) on the renin-angiotensin system (RAS) and the kallikrein/kinin system (KKS)

9) Silent information regulator 1 (SIRT-1, Sirtuin 1): SIRT1, a protein deacetylase dependent on NAD^+^, is engaged in several biological processes, like cell death, inflammation, response to oxidative stress, and metabolism by regulating principal targets via deacetylation (63). The increase in the NAD^+^/NADH ratio leads to SIRT-1 activity. One of the downstream targets of SIRT1 is the peroxisome proliferator-activated receptor-γ coactivator (PGC-1α). SIRT-1 cleaves the acetyl groups from the PGC-1α protein in a reaction that generates nicotinamide (NAM) and 2’-O-acetyl-ADP-ribose in order to activate it. The deacetylated PGC-1α protein promotes the anti-oxidant protective response (64). The reduction in the activity of SIRT-1 would attenuate the deacetylation rate of PGC-1α. Hyperacetylation declines the activity of PGC-1α which leads to reduced mitochondrial respiratory capacity and ROS detoxification (65). In SARS-CoV-2 infection, due to the blocking of complex IV, as mentioned, the amount of NAD^+^ decreases, and the SIRT-1/PGC-1α pathway would be inhibited. MB may improve the anti-oxidant effect of this pathway by increasing the SIRT-1 activity via boosting the NAD^+^/NADH ratio or affecting post-transcriptional regulatory miRNAs (Figure 1 M) (66). 

10) Nrf2-ARE signaling pathway: Nuclear erythroid 2-related factor 2 (Nrf2), a basic domain-leucine zipper (bZlP) transcription factor, through interaction with the anti-oxidant response element (ARE) regulates the expression of a plethora of genes involved in the anti-inflammatory and anti-oxidant defense as well as mitochondrial protection (67). Furthermore, the activation of Nrf2 down-regulates the ACE2 expression which is needed to enter SARS-COV-2 in the host cell (68). Recently, it has been shown that the expression pathway of Nrf2 is inhibited in biopsies obtained from COVID-19 patients (69). Methylene blue can indirectly cause the activation of Nrf2, in such a way that by accepting electrons from NADH, methylene blue leads to an increase in the NAD^+^/NADH ratio. This brings about AMP-activated protein kinase (AMPK) activation, which can activate the Nrf2/ARE signaling pathway via phosphorylation of Nrf2 (70). Moreover, H_2_O_2_ produced in the electron transport chain due to MB treatment can prompt dissociation between Nrf2 and KEAP1 which is necessary for the activation of Nrf2 (Figure 1 N) (70). 

11) Dysregulation of the renin-angiotensin system (RAS) occurs during SARS-CoV-2 infection. The RAS affects fluid homeostasis and the cardiovascular system. The RAS cascade starts from angiotensinogen (AGT) that is cleaved by renin (REN) and the peptide angiotensin I (Ang I) is liberated. Then, two amino acids from Ang I are removed by the angiotensin-converting enzyme (ACE) to form the multi-functional peptide angiotensin II (Ang II). Ang II performs its main functions by binding to angiotensin type 1 (AT1R) and type 2 (AT2R) receptors. ACE2 converts Ang I into angiotensin 1-9 (Ang-(1–9)) and Ang II into angiotensin 1-7 (Ang-(1–7)). Afterward, Ang-(1–9) is changed to Ang-(1–7) by ACE. Ang-(1–7) through interaction with Mas receptor (MasR) exerts anti-fibrotic, anti-oxidant, anti-inflammatory, antithrombotic, and vasodilatory properties and diminishes the effects of vasoconstriction, thrombosis, and inflammation of ACE/Ang II/AT1R axis (71). In addition, it can also activate the bradykinin (BK)-nitric oxide (NO) cascade by binding to AT2R in order to produce NO which promotes the β-arrestin pathway that stimulates AT1R desensitization and internalization of the receptor (72). Hence, the proper expression and function of ACE2 are vital to the balance of RAS and protection from cell injury. In the SARS-COV-2 infection, ACE2 expression and its enzymatic activity are reduced due to binding the virus to ACE2 receptors for entrance into the host cells (73). Thus, an increase in the level of Ang II occurs as a result of reduction in the ACE2 expression. The increased Ang II level stimulates the signaling pathways of vasoconstriction, inflammation, and coagulation through the Ang II/AT1R axis. By way of illustration, the activated Ang II/AT1R axis prompts the plasminogen activator inhibitor 1 (PAI-1) and tissue factor (TF) expression in diverse cells, which increases the risk of thrombosis and coagulation (73). Methylene blue can block the interaction between the SARS-CoV-2 S protein and ACE2 receptor and improves dysregulation of RAS during SARS-CoV-2 infection (3, 7, 28) (Figure 2 A). 

12) Dysregulation of the Kinin-Kallikrein System (KKS)/bradykinin storm/vasoactive peptide storm: KKS, an endogenous multiprotein cascade that controls the inflammation, blood pressure, coagulation, and pain pathways, appears to be disrupted in COVID-19. Bradykinin (BK or BK-1-9) is one of the important mediators of this cascade that plays a crucial role in vasodilation and inflammation in many cell types. In inflammation of COVID-19, BK production is increased due to potentiation of high-molecular-weight kininogen (HK) cleavage by kallikrein (KAL). BK can bind to the G protein-coupled bradykinin B2 receptor (BKB2R or B2R) and activates signaling pathways involved in increased vasodilation, inflammation, pain, edema formation, and vascular permeability. Furthermore, some of the BK may be transformed into [des-Arg9]-BK (BK-1-8 or DABK)), an active metabolite, by Kininase І (carboxypeptidase N) (71). DABK acts as a G protein-coupled bradykinin B1 receptor (BKB1R or B1R) agonist and a substrate of ACE2. Although in normal conditions ACE2 inactivates DABK and attenuates the BKB1R signaling pathway, in COVID-19 disease the ACE2 depletion in the cell surface and the increased level and activity of DABK occur due to virus internalization (74, 75). More activation of DABK/BKB1R axis signaling during COVID-19 contributes to exaggerated inflammation, vasodilation, leukocyte extravasation, angioedema, nitric oxide production, arachidonic acid release, and acute respiratory distress syndrome (ARDS) development (71, 75). 

Increased production of nitric oxide induces relaxation in vascular smooth muscle cells (VSMC). The activation of the G-proteins coupled with BKB2R stimulates phospholipase C β (PLCβ), which cleavages phosphatidylinositol 4,5-bisphosphate (PIP_2_) to diacylglycerol (DAG) and inositol 1,4,5-trisphosphate (IP_3_). IP_3_ attaches to its receptor in the endoplasmic reticulum (ER) and boosts Ca^2+^ extrusion. The Ca^2+^ linkage to calmodulin promotes eNOS activation which triggers the NO synthesis. NO diffuses to VSMC and attaches to and activates soluble guanylyl cyclase (sGC). It converts guanosine triphosphate (GTP) to cyclic guanosine monophosphate (cGMP) which activates the cGMP-protein kinase G (PKG), which subsequently leads to the relaxation of VSMC (72). 

In addition, BK binding to BKB2R also activates protein kinase C (PKC) and consequently cytosolic phospholipase A_2_ (cPLA_2_) which causes arachidonic acid to hydrolyze from PIP_2_. The cyclooxygenase 1 (COX-1) and cyclooxygenase 2 (COX-2) enzymes convert arachidonic acid to prostaglandin H_2_ (PGH_2_). Prostaglandin I_2_ or PGI_2_ (prostacyclin) and thromboxane A2 (TXA2) are derived from PGH_2_ via the action of some synthases. PGI_2_ inhibits platelet activation and has vasodilatory properties but TXA2 has the opposite characteristics (76). 

MB may eliminate the unfavorable effects of bradykinin storm by inhibiting nitric oxide synthase, guanylyl cyclase enzyme, and binding of viral S protein to ACE2 receptors (Figure 2 A and B) (31, 77).

13) COVID–19–associated mucormycosis (CAM): Mucormycosis is a life-threatening opportunistic angioinvasive fungal infection that increases the possibility of morbidity and mortality in COVID-19 patients. MB can damage the fungal mitochondria at a concentration of 500 ppm and may be very efficient in the treatment of CAM (12). 

**Studies on the use of MB against SARS-CoV-2**

***Clinical studies***

In 2020, Naymagon *et al*. reported the results of treatment with MB (1–2 mg/kg) in 3 COVID-19 patients with significant methemoglobinemia. After receiving MB, the level of methemoglobin (Met-Hb) increased in all 3 patients, and one patient was diagnosed with G6PD deficiency (79). 

In 2020, researchers described that hypoxia and methemoglobinemia in a patient undergoing treatment for COVID-19 pneumonia did not respond to intravenous MB (1.5 mg/kg) treatment and the administration of packed red blood cell transfusions was required (80).

In 2020, Henry *et al*. mentioned antiviral properties and possible protective role of MB against respiratory viruses based on a retrospective study on 2500 French cancer patients treated with a combination of α-lipoic acid (800 mg twice a day), hydroxycitrate (500 mg three times daily), and MB (75 mg three times a day). Because none of the study subjects developed influenza-like illness during the COVID-19 epidemic (2).

In 2020, researchers administered MB (1 mg/kg) along with vitamin C (1500 mg/kg) and N-acetyl Cysteine (1500 mg/kg) (MCN) orally or intravenously for the five hospitalized COVID-19 patients in ICU as the last therapeutic option (ClinicalTrials.gov Id: NCT04370288, IRCT Id: 49,767). Four of the five patients showed marked improvement in symptoms (decrease in nitrite, nitrate, methemoglobin, and oxidative stress) which increased the possibility of MCN ‘s positive effect in treating and the survival rate of these patients (16).

In 2020, Strakhovskaya *et al*. used oral MB (1 mg/kg water solution) and photodynamic therapy (PDT 650 nm) to treat 60 people with COVID-19. Using MB-PDT completely inhibited the initial virus titers 10^6^/ml and 10^7^/ml. These results indicated the advantages of MB combined with light sources in the inactivation of pathogens (81). 

In 2021, a study used a combination of PDT (660 nm 32.14 J/cm -808 nm with 0.01 % MB photosensitizer) and photobiomodulation therapy (PBMT) for extensive lip lesions in a patient suffering from COVID-19. He was completely healed within around four days. According to this study, a combination of PDT and PBMT is a promising method for the management of lip lesions associated with COVID-19 (82).

In 2021, researchers also demonstrated the therapeutic benefit of the reduced form of methylene blue (leucomethylene) in a phase 2 clinical trial study of patients with severe COVID-19. The authors randomly assigned patients to receive standard of care (SOC), plus the syrup containing MB, vitamin C, dextrose, and N-acetyl cysteine (n = 40), or to receive only SOC (n = 40). In the syrup-receiving group, a significant improvement in oxygen saturation (SpO_2_) and respiratory rate (RR) and a considerable decrease in hospital stay and mortality were observed, but not in the SOC group (83).

In 2021, Hamidi-Alamdari *et al*. reported the results of their phase 3 clinical trial about the efficacy of MB (the reduced form) as an adjunct therapy in the treatment of COVID-19 patients. Two hundred twenty-three hospitalized patients with laboratory-confirmed severe COVID-19 were randomly assigned to receive standard care along with the syrup that contained MB, vitamin C, dextrose, and N-acetyl cysteine (n = 106) or to receive standard care (SC) alone (n = 117). This trial showed that the length of hospitalization and mortality in the syrup-receiving group were significantly decreased compared to the group receiving only standard care (10).

In 2021, Lotfabadi *et al*. in their case series report applied a standard care protocol along with the MB syrup similar to Hamidi-Alamdari *et al*.’s study (82) to save 83 COVID-19 patients who had not responded to remdesivir, interferon-β, and favipiravir therapies. Seventy-two patients recovered entirely, and eleven patients did not survive (84).

In 2021, a study used the syrup containing MB prepared by Hamidi-Alamdari* et al*. (82) for ten severe COVID-19 patients who failed to respond to remdesivir, interferon-β, and favipiravir (1 mg/kg every 8 hr for two days, followed by 1 mg/kg every 12 hr for the next days). All patients recovered completely (85). 

In 2021, researchers conducted a case series study on 50 hypoxic COVID-19 patients (SpO_2_/FiO_2_ <200) who were treated with intravenous MB apart from the standard of care (in a dosage of 1 mg/kg body weight, with a maximum of five doses). After MB injection the median SpO_2_/FiO_2_ ratio and ventilator-free days progressively improved and the proinflammatory biochemical parameter was significantly reduced. Thirty subjects were discharged with a 60% recovery rate while twenty patients succumbed to the disease (23).

In 2022, Lotfabadi *et al*. administered the MB syrup along with standard care to seven outpatients with confirmed cases of severe COVID-19 to verify its effectiveness. All outpatients were healed (86).

In 2022, a study investigated the effect of using intravenous infusion of 250–300 ml of high anti-SARS-CoV-2 IgG titers methylene blue-treated convalescent plasma in the treatment of outpatients with mild COVID-19 symptoms in a randomized, placebo-controlled trial (250 ml of sterile 0·9% saline solution) in 4 health-care centers in Spain (ClinicalTrials.gov identifier: NCT04621123). Three hundred seventy-six participants were randomly assigned to two groups to treat (convalescent plasma n=188 [serum antibody-negative n=160]; placebo n=188 [serum antibody negative n=166]). The results showed that the mean change in viral load, the average hospitalization time, and the progression rate of the disease from mild to severe did not differ in the two groups after intervention. As a result, the use of methylene blue-treated convalescent plasma to treat COVID-19 outpatients is not effective (87).

In 2022, researchers evaluated the effect of nebulized methylene blue (NMB) on the clinical course of 63 RT-PCR-confirmed COVID-19 cases. The participants were divided into three groups of 21 people, whose treatment plans were such that the first group received 0.5 mg NMB along with1.25 mg bronchodilator levosalbutamol plus 500 mcg ipratropium three times a day, the second group received 0.5 mg NMB along with 1 mg inhalational steroid budesonide, and the third group as control did not receive NMB in their treatment plan. A general trend of reduced hospital stays, inflammatory markers, and oxygen requirements in groups receiving NBE (groups 1 and 2) vs control (group 3) was observed (18).

In 2022, a prospective, single-center study administrated MB for 103 cases with ARDS due to COVID-19 infection. The administration route of MB was through NMB (0.5 ml of 0.5% MB solution + 2.5 ml of distilled water) 3 times a day as long as the case was admitted. An ampoule of 10 ml of MB plus 90 ml of potable water through an oxygen port was prescribed until the patient needed oxygen. MB (a dosage of about 2 mg/kg body weight) in 300 ml of normal saline (NS) over 3 hr was given once a day for 5 days. Upon MB treatment, the levels of serum ferritin, lactate dehydrogenase, D-dimer, and C-reactive protein decreased compared to before administration. The administration of MB accelerated patients’ recovery by controlling the hyperimmune response and improving oxygen saturation (88). 

In 2022, researchers evaluated the effect of local viral photodynamic inactivation (49 J/cm2 for 5 min) of 7.5 ml of 1% MB solution (MB-PDI) in the oral and nasal cavities in combination with the oral-MB (1.5 to 2 mg/kg 12-hourly) and/or photobiomodulation (PBM) (4.9 J/cm2) for eight individuals with the disease. Significant clinical improvement without side effects occurred mostly within 12–24 hr after starting the treatment protocol in patients. None of the subjects who completed the protocol required hospitalization or show re-infection with COVID-19 during the one-year follow-up. Their results showed that this protocol may be a safe and promising approach to challenge the COVID-19 disease (89). 

In 2022, a study used antimicrobial PTD (aPTD) (0.01% MB, low-laser 4 J, 100mW, 660 nm) with intravenous acyclovir or topical penciclovir for herpes-like lesions in the lips and oral mucosa of 4 patients with severe COVID-19. Rapid recovery was observed in 3 cases (90).

In 2022, 64 patients were treated with moderate symptoms of COVID-19 (32 pregnant women and 32 subjects of the general population) with 1 ml of 0.1% MB+ budesonide nebulization in 0.9% normal saline/ distilled water, Tab Cefditoren Pivoxil (400 mg), twice daily for 5 days, without any untoward sequelae (91). 

In 2022, Franzen reduced the viral load of COVID-19 in the upper airways in a married couple with a combination of 0.001% methylene blue solution and a simple LED flashlight with more than 5 log steps. The couple recovered five to six days after the onset of symptoms. As a result, photodynamic inactivation using MB is an inexpensive and simple treatment method for the early phase of SARS-CoV-2 infection without involving the deeper bronchial parts or systemic spread of the virus in the body (92).

In 2022, a proof-of-principle clinical trial (42 patients) investigated the therapy effect of MB-aPDT (0.01% MB, 670 nm) to the anterior nares for early stages of COVID-19 by virological quantification via RT-qPCR and infectivity assay. The infectivity assay showed a viral reduction in 90% of samples and complete viral inactivation in 70% of them after one session of aPDT (93).

In 2023, a study used aPDT (0.01% MB, 660 nm, 100 mW, 32.14 J/cm2, 6 J, and 60 sec per point) to inactivate SARS-CoV-2 in orofacial lesions. A slightly remarkable improvement in phlogistic symptoms and spontaneous bleeding of the orofacial lesions was observed within 24 hr (94).

In 2023, research used MB nebulization intervention (5 ml of 10 ml solution of MB in 200 ml of distilled water) twice a day as a prophylactic agent in preventing mucormycosis associated with COVID-19. The results did not show a significant benefit in the use of MB in the prevention of mucormycosis associated with COVID-19 (95). 

In addition, two trials have documented the initiation of MB use for COVID-19 treatment (according to ClinicalTrial.gov): one in Mexico (NCT04619290; sublingual MB with 50 min low-level light therapy. Up to 7 days) and one in Switzerland (NCT04635605; 100 mg MB two times a day for 5 days) but their printed results are as yet not available.

A. MB is a virucidal agent inhibiting the interaction of ACE2 and S protein. B. MB blocks the viral attachment by accumulating on HSPGs. C. MB inhibits phagosome maturation by increasing the endosome's intracellular pH. D. MB blocks translation and transcription as a result of interaction with RNA. E. MB prevents cytokine storm by inhibiting priming and activation of NLRP3 inflammasome. F. MB inhibits apoptosis and pyroptosis by hindering caspases. G. MB restores the function of the T cells by blocking their downstream inhibitory protein-protein interaction. H. MB decreases iNOS by inhibiting the binding of NF-ĸβ. I. MB directly inhibits iNOS and leads to reduction in hypoxemia and oxidative stress as well as the rescue of mitochondrial function. J. MB reduces hypoxemia by changing MetHb to Hb. K. MB functions as an alternative electron transfer with ETC to rescue mitochondrial function. L.MB. reduces ROS by inhibiting xanthine oxidase. M. MB activates the SIRT1/PGC-1α pathway by increasing NAD+. The schematic of the “NLRP3 inflammasome” was taken from the article of Yap *et al* (78).

A. MB improves the dysregulation of RAS and KKS by blocking the interaction between the S protein and the ACE2 receptor. B. MB eliminates the unfavorable effects of bradykinin storm by inhibiting nitric oxide synthase (NOS) and soluble guanylyl cyclase enzyme (sGC).

**Table 1:** Summary of different studies evaluating the role of methylene blue (MB) in COVID-19

No.	Year of publication	Study design	Methylene blue intervention summary	Results in brief	References
**1**	2020	Case report	1–2 mg/kg	After receiving MB, the level of methemoglobin increased in all 3 patients, and one patient was diagnosed with G6PD deficiency	(78)
**2**	2020	Case report	1.5 mg/kg	Patient did not respond to intravenous MB treatment	(79)
**3**	2020	Retrospective study	75 mg three times a day	2500 French cancer patients treated with a combination of α-lipoic acid, hydroxycitrate, and MB did not develop influenza-like illness during the COVID-19 epidemic	(2)
**4**	2020	RCT	1 mg/kg	Four of the five patients showed marked improvement in symptoms which increased the possibility of MB's positive effect in treating and the survival rate of these patients	(16)
**5**	2020	RCT	1 mg/kg	Results indicated the advantages of MB combined with light sources in the inactivation of pathogens	(80)
**6**	2020	*In vitro*	Not applicable	THERAFLEX MB-Plasma (MB/light) effectively reduces the infectivity of SARS-CoV in plasma.	(30)
**7**	2020	*In vitro*	0.1–100 μM for 48 h at 37 °C	MB with an inhibitory concentration (IC) at a very low micromolar range (IC50= 0.3 μM and IC90= 0.75 μM) has an antiviral activity more effective and higher than oral uptake and I.V. administration	(9)
**8**	2020	*In vitro*	3 μM	MB on the viral attachment and entry of SARS-CoV-2 into the host cell by blocking the interaction of the viral spike protein with the ACE2 protein on the host cell	(3)
**9**	2021	Case report	0.01 % solution	Extensive lip lesions in a patient suffering from COVID-19 were completely healed within around four days	(81)
**10**	2021	RCT	14 mg/ml MB in 50% sucrose	A significant improvement in oxygen saturation and respiratory rate and a considerable decrease in hospital stay and mortality were observed	(82)
**11**	2021	RCT	1 mg/kg every 8 hr for two days, followed by 1 mg/kg every 12 hr for the next 12 days	Length of hospitalization and mortality were significantly decreased in the intervention group compared to the control group	(10)
**12**	2021	Case series	14 mg/ml MB in 50% sucrose	72 COVID-19 patients who had not responded to remdesivir, interferon-β, and favipiravir therapies recovered entirely, and 11 patients did not survive	(83)
**13**	2021	Case series	1 mg/kg every 8 hr for two days, followed by 1 mg/kg every 12 hr for the next 12 days	All ten patients recovered completely	(84)
**14**	2021	Case series	A dosage of 1 mg/kg body weight, with a maximum of five doses	30 subjects were discharged with a 60% recovery rate while 20 patients succumbed to the disease	(23)
**15**	2021	RCT	1 mg/kg three times a day for 2 days, followed by 1 mg/kg two times a day for the next 12 days	All seven outpatients were healed	(85)
**16**	2021	*In* *vitro*	Different dilutions of MB (2 µg/ml (6.25 µM), 0.4 µg/ml (1.25 µM), 0.08 µg/ml (0.25 µM)) for 2 h or 20 h	Virucidal preventive or therapeutic activity of MB at low micromolar concentrations and in the absence of UV activation against SARS-CoV-2 viruses was shown	(19)
**17**	2021	*In* *vitro*	0.1 µM to 100 µM	MB had a concentration-dependent antiviral activity against two SARS-CoV-2 strains (IHUMI-3 and IHUMI-6) and also interacted at both stages of entry and post-entry of virus infection in the cells	(6)
**18**	2021	*In vitro*	0.22 μg/ml	MB and Radchlorin had *in* *vitro* viral activity even without light-induced activation, but their effectiveness with PDT was higher, faster, and at lower concentrations	(1)
**19**	2022	A randomized, placebo-controlled trial	Not applicable	Use of methylene blue-treated convalescent plasma to treat COVID-19 outpatients is not effective	(86)
**20**	2022	An observational study	0.5 mg nebulized methylene blue (NMB), three times a day	A general trend of reduced hospital stays, inflammatory markers, and oxygen requirements in groups receiving NBE vs control was observed.	(18)
**21**	2022	A prospective, single-center study	NMB (0.5 ml of 0.5% MB solution+ 2.5 ml of distilled water) three times a day, MB (a dosage of about 2 mg/kg body weight) in 300 ml of normal saline over 3 hr was given once a day for 5 days	Administration of MB accelerated patients’ recovery.	(87)
**22**	2022	Pilot Study	Viral photodynamic inactivation of 7.5 ml of 1% MB solution and oral-MB (1.5 to 2 mg/kg 12-hourly)	Protocol may be a safe and promising approach to challenge the COVID-19 disease	(88)
**23**	2022	Case report	Antimicrobial PTD with 0.01% MB	Rapid recovery was observed in 3 out of 4 patients	(89)
**24**	2022	RCT	1 ml of 0.1% MB	Combination of 1 ml of 0.1% MB+ budesonide nebulization in 0.9% normal saline/ distilled water, Tab Cefditoren Pivoxil (400 mg), twice daily for 5 days was effective in treating COVID-19-affected pregnant women and the general population safely, without any untoward sequelae.	(90)
**25**	2022	Case report	0.001% MB solution	Photodynamic inactivation using MB is an inexpensive and simple treatment method for the early phase of SARS-CoV-2 infection	(91)
**26**	2022	A proof-of-principle clinical trial	0.01% MB solution	Infectivity assay showed a viral reduction in 90% of samples and complete viral inactivation in 70% of them after one session of therapy	(92)
**27**	2022	*In vitro*	LED flashlight with 0,001% and 0,0001% MB solution	Photodynamic inactivation based on MB resulted in a 99.99% reduction of intracellular and extracellular SARS-CoV-2 virus after one minute of exposure	(95)
**28**	2022	*In vitro*	20 µl of different dilutions of MB (0, 0.01, 0.1, 1 and 10 μM)	MB can inactivate all SARS-CoV-2 viruses on respirators	(96), (97)
**29**	2022	*In vitro*	Different dilutions of MB (0, 0.01, 0.1, 1 and 10 μM) with light (MBL)	MBL can inactivate 99.8 to 99.9% of all coronaviruses in N95 filtering facepiece respirators and medical masks without compromising their integrity during 5 decontamination cycles	(98)
**30**	2022	*In vitro*	10 μM	MB blocks infection of primary human nasal airway epithelial explant cultures with Wuhan strain, and five variants of Alpha (B.1.1.7), Beta (B.1.351), Gamma (B.1.1.28), Delta (B.1.617.2), and Omicron BA.1 (B.1.1.529.1)	(99)
**31**	2022	*In vitro*	10 μM	There was a significant reduction in viral load up to~ 6 Logs in all samples treated with MB-light compared to control groups	(92)
**32**	2023	Case report	0.01% MB solution	A slightly remarkable improvement in phlogistic symptoms and spontaneous bleeding of the orofacial lesions was observed within 24 hr	(93)
**33**	2023	RCT	5 ml of 10 ml solution of MB in 200 ml of distilled water, twice a day	Results did not show a significant benefit in the use of MB in the prevention of mucormycosis associated with COVID-19	(105)
**34**	2023	In vitro	⁓0.8 μmol/L	Treatment of spiked plasma with the THERAFLEX MB-Plasma system can be an effective option to reduce the risk of transmission of emerging pathogens through blood transfusion	(100)

***In vitro studies***

In 2020, THERAFLEX MB-Plasma pathogen inactivation system was used to inactivate the SARS-CoV in plasma and platelet concentrate (PC) samples. The results showed that the mentioned system effectively inactivated the SARS-CoV spike in the samples (30).

In 2020, it was demonstrated that non-photoactivated MB with an inhibitory concentration (IC) at a very low micromolar range (IC_50_= 0.3 μM and IC_90_= 0.75 μM) has an antiviral activity more effective and higher than drugs such as ritonavir (>100 μM), lopinavir (26.6 μM), remdesivir (23 μM), azithromycin (20.1 μM) and hydroxychloroquine (1.5 μM), against SARS-CoV-2 (strain IHUMI-3) in *in vitro* condition (9).

In 2020, researchers reported the inhibitory effect of MB (IC_50_= 3 μM) on the viral attachment and entry of SARS-CoV-2 into the host cell by blocking the interaction of the viral spike protein with the ACE2 protein on the host cell *in vitro* (3).

In 2021, a study showed the virucidal preventive or therapeutic activity of MB at low micromolar concentrations and in the absence of UV activation against SARS-CoV-2 viruses. Considering the antiviral activity of MB, even without UV activation, although with slower kinetics, they also confirmed that its antiviral activity is based on several mechanisms of action. Finally, they proved the additive effect of MB when administered simultaneously with immune sera, which was specifically due to the presence of immunoglobulins against SARS-CoV-2 (19). 

In 2021, the antiviral activity of MB against two SARS-CoV-2 strains (IHUMI-3 and IHUMI-6) were investigated alone and in combination with other potential drugs and its effects on the entry and the post-entry of the virus into Vero E6 cells. MB had a concentration-dependent antiviral activity against both investigated strains and also interacted at both stages of entry and post-entry of virus infection in the cells. The effects of MB in combination with quinine, mefloquine, and pyronaridine drugs were additive, but in combination with chloroquine, hydroxychloroquine, desethylamodiaquine, piperaquine, lumefantrine, ferroquine, dihydroartemisinin, and remdesivir they were antagonist (6).

In 2021, researchers evaluated the use of photodynamic therapy (PDT, 662 nm 16 J/cm^2^-40 J/cm^2^) with photosensitizers (PSs) (MB and Radachlorin) to inactivate SARS-CoV-2 and inhibit its replication within the SARS-CoV-2-infected green monkey kidney cells and viral suspensions. The results confirmed the antiviral effect of PDT in the presence of MB and Radachlorin in viral suspension and infected cells. MB and Radchlorin had *in vitro* viral activity even without light-induced activation, but their effectiveness with PDT was higher, faster, and at lower concentrations (1).

In 2022, Arentz and von der Heide used local antiviral photodynamic inactivation (PDI) based on MB to reduce the viral load in the nose and throat area in the early phase of a COVID-19 infection. The result of their study was a promising, inexpensive, and easy method of combination: a white LED lamp and a low concentration of MB to treat COVID-19 in the early phage. This method resulted in a 99.99% reduction of intracellular and extracellular SARS-CoV-2 virus after one minute of exposure (96).

In 2022, considering the COVID-19 pandemic and the shortage of N95 respirators around the world, researchers used photoactivated MB to sterilize them for reuse. The findings showed that photoactivated MB can inactivate all SARS-CoV-2 viruses on respirators (97). After disinfection, the exposure risk to inhaling MB when reusing face masks was very low and at the threshold of safety (98).

In 2022, a study assessed the effect of MB with light (MBL) decontamination treatment on 3 N95 filtering facepiece respirators (FFR) and 2 medical mask models infected with the coronavirus. The equipment was treated with 10 μM MB and exposed to 50,000 lux of white light or 12,500 lux of red light for 30 min. The results showed that MBL can inactivate 99.8 to 99.9% of all coronaviruses in FFRs and medical masks without compromising their integrity during 5 decontamination cycles (99). 

In 2022, a study reinforced the anti-coronavirus effects of MB. MB (10 μM) blocked infection of primary human nasal airway epithelial explant cultures (HAEEC) with Wuhan strain, and five variants of concern (VoC), Alpha (B.1.1.7), Beta (B.1.351), Gamma (B.1.1.28), Delta (B.1.617.2), and Omicron BA.1 (B.1.1.529.1) (100).

In 2022, the effect of MBPDT (10 min incubation at 10 μM and 30 J cm^−2^) to inactivate SARSCoV2 in six samples derived from COVID-19 patients was investigated. There was a significant reduction in viral load up to~ 6 Logs in all samples treated with MB-light compared to control groups (93).

In 2023, considering the therapeutic importance of convalescent plasma as well as the possibility of the risk of transmission of emerging pathogens through it, researchers investigated the ability of the THERAFLEX MB-Plasma system to inactivate SARS-CoV-2 of convalescent plasma. SARS-CoV-2 spiked plasma units were treated using this system in the presence of MB (⁓0.8 μmol/L; visible light doses: 20, 40, 60, and 120 [standard] J/cm2). The results showed that treatment of spiked plasma with the THERAFLEX MB-Plasma system can be an effective option to reduce the risk of transmission of emerging pathogens through blood transfusion (101).

According to the studies, MB can simultaneously affect most of the host’s harmful responses caused by the SARS-CoV-2 infection due to its multiple properties, including anti-hypoxemia, anti-oxidant, immune system modulator, and antiviral. Interestingly, based on some discussed papers in this review, MB can also prevent SARS-CoV-2 infection (Table 1).

## Conclusion

Methylene blue, due to polypharmacology in action and multiple properties, can simultaneously affect most of the host’s harmful responses caused by the SARS-CoV-2 infection including severe hypoxia, hyperinflammatory reactions, and oxidative stress, and therefore can be used alone or in combination with other drugs as a safe, effective, inexpensive, accessible treatment option with minimal side effects in the prevention and clinical management of COVID-19 patients. 

## Authors’ Contributions

E E and DHA designed the study; E E and S A wrote the original draft; HAD and D A reviewed and edited the manuscript. All authors read and approved the final manuscript.

## Conflicts of Interest

None.
